# Swarm Intelligence for Collaborative Play in Humanoid Soccer Teams

**DOI:** 10.3390/s25113496

**Published:** 2025-05-31

**Authors:** Farzad Nadiri, Ahmad B. Rad

**Affiliations:** Autonomous and Intelligent Systems Laboratory, School of Mechatronic Systems Engineering, Simon Fraser University, Surrey, BC V3T 0A3, Canada; farzad_nadiri@sfu.ca

**Keywords:** humanoid soccer robots, swarm intelligence, decentralized control, robot communication

## Abstract

Humanoid soccer robots operate in dynamic, unpredictable, and often partially observable settings. Effective teamwork, sound decision-making, and real-time collaboration are critical to competitive performance. In this paper, a biologically inspired swarm-intelligence framework for humanoid soccer is proposed, comprising (1) a low-overhead communication User Datagram Protocol (UDP) optimized for minimal bandwidth and graceful degradation under packet loss; (2) an Ant Colony Optimization (ACO)-based decentralized role allocation mechanism that dynamically assigns attackers, midfielders, and defenders based on real-time pheromone trails and local fitness metrics; (3) a Reynolds’ flocking-based formation control scheme, modulated by role-specific weighting to ensure fluid transitions between offensive and defensive formations; and (4) an adaptive behavior layer integrating lightweight reinforcement signals and proactive failure-recovery strategies to maintain cohesion under robot dropouts. Simulations demonstrate a 25–40% increase in goals scored and an 8–10% boost in average ball possession compared to centralized baselines.

## 1. Introduction

**Figure 1:**
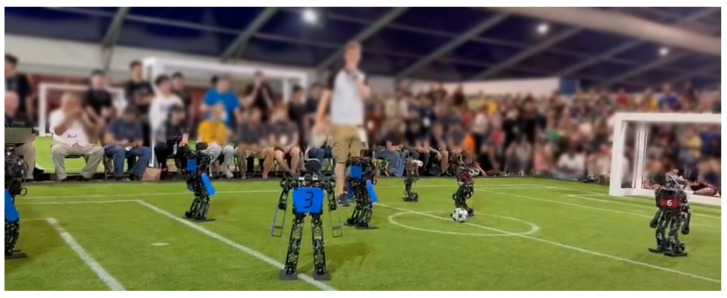
Final match of the child-sized humanoid soccer RoboCup2024 between CIT Brains and KURA.

RoboCup Soccer (Figure 1) has served as a premier benchmark for multi-agent and humanoid robotics over the past two decades, offering a dynamic testbed for advanced perception, locomotion, and decision-making [1,2,3]. In the same spirit as the human soccer team strategy, effective team coordination is central in humanoid soccer. This demands task allocation, distributing roles, sharing situational awareness, and executing adaptable formations in the face of rapid, often volatile situations on the field [4,5]. Traditional centralized strategies, which typically rely on global vision systems or a central server, have shown effectiveness under controlled conditions [6]. However, they introduce communication bottlenecks, latency, single-point failures, and poor scalability as the number of robots grows [7].

In order to alleviate these shortcomings, a variety of distributed and decentralized methods have emerged in robotic soccer research, including multi-agent reinforcement learning [8] and consensus-based algorithms [9]. Although these approaches reduce dependence on a single centralized node, many still rely on partial global knowledge or extensive offline training, limiting their real-time adaptability. Methods for decentralized role assignments, improve resilience but could struggle to handle continuous disruptions that are common in fast-paced matches. In recent studies, the RoboCup Standard Platform League has experienced a substantial decrease in network packet rates, which challenges the conventional coordination methods. Consequently, novel distributed architectures have been proposed to enable efficient multi-agent coordination under limited communication scenarios [10]. Recent developments have demonstrated that robust team play can be achieved with minimal inter-robot communication, as evidenced by the B-Human framework, which leverages local decision-making to mitigate communication bottlenecks [11]. A comprehensive survey of game strategies for physical robot soccer highlights the effectiveness of decentralized control systems and provides valuable insights into emerging coordination techniques [12].

This paper introduces a swarm-based control framework specifically designed for humanoid soccer robots. The framework aims to design a robust communication protocol that supports distributed decision-making while minimizing latency. It seeks to develop a dynamic role allocation mechanism inspired by Ant Colony Optimization [13] to enhance multi-robot collaboration. The framework incorporates flocking-based formation control [14] to ensure smooth transitions between offensive and defensive strategies. Additionally, it aims to enable adaptive behaviors through real-time learning and proactive failure management, ensuring resilience. The proposed approach will be validated in simulation platforms, providing a comprehensive analysis of its strengths and limitations.

### 1.1. Swarm Intelligence in Multi-Robot Systems

Swarm intelligence offers a compelling alternative through fully decentralized control and emergent coordination of agents. The integration of swarm intelligence within cyber-physical systems has been shown to significantly improve the robustness and scalability of multi-robot operations by leveraging local interactions to achieve global objectives [15]. This theoretical foundation supports the use of Ant Colony Optimization (ACO) for role allocation and Reynolds’ flocking [16] for formation control. Inspired by collective behaviors in nature—such as ant colonies, bird flocks, and fish schools—swarm systems operate without a central authority, relying on local interactions to achieve global objectives [17]. In multi-robot contexts, swarm principles have proven effective for tasks like autonomous navigation, formation control, and sensor coverage [18]. Nonetheless, most swarm-based applications focus on wheeled robots or simpler tasks, leaving humanoid soccer—where bi-pedal locomotion and rapid adaptation are paramount—relatively underexplored.

Adopting swarm intelligence for humanoid soccer teams can mitigate many disadvantages associated with the centralized architecture. Humanoid soccer robots are often equipped with limited onboard vision, and they generally do not have complex locomotion; as such, they benefit from local decision-making and asynchronous coordination. As each robot continuously adjusts its balance and stepping patterns, swarm-based rules allow essential information (e.g., ball position, respective teammate locations) to propagate through minimal local exchanges, thus reducing the need for a global field view. Moreover, sudden disturbances such as ball deflections or collisions are handled more gracefully when the system can adapt via decentralized, emergent strategies rather than waiting on instructions from a central controller.

### 1.2. Decentralized Control and Emergent Coordination

Researchers have long recognized the importance of decentralized control in multi-robot tasks prone to dynamic changes [19]. In robotic soccer, both graph-based and potential field methods have been explored to maintain formations or guide positioning [20,21]. While these methods can provide structure, they often lack flexibility in the face of rapid ball turnovers or unexpected role switches.

Emergency coordination through machine learning has also gained attention in the last few years. Multi-agent reinforcement learning (MARL) approaches can teach complex interactions like ball passing or opponent tracking [22]. However, the high data requirements and offline training phases make them less responsive to real-time changes in the field. By contrast, swarm-based coordination enables continuous role adjustments driven by localized interactions, preserving adaptability even when global conditions shift abruptly. Recent advances in multi-agent reinforcement learning (MARL) have produced domain-specific frameworks that achieve high-quality team play in robot soccer. Orr and Dutta’s 2023 survey of deep MARL for multi-robot coordination highlights scalable policy learning under limited communication and sample efficiency in real-world setups [8]. Brandão et al. (2022) demonstrate a self-play MARL approach, achieving strategic decision-making in 11v11 simulated soccer [23]. Pu et al. (2024) present a systematic review of how artificial-intelligence techniques—especially deep learning, multi-agent reinforcement learning, and other data-driven models—are being applied to orientation (situational awareness from event- and tracking-data) and decision-making (tactical choice, action selection) in soccer [24]. Li et al. (2024) introduce MARLadona, a decentralized MARL pipeline on an Isaac Gym-based soccer environment that—using only local observations, curricula, and self-play—achieves high win rates against state-of-the-art heuristic agents while supporting true online adaptation [25].

In contrast, the proposed biologically inspired ACO + flocking framework operates with only lightweight local interactions and yields sub-second reaction times and minimal data requirements in live humanoid soccer.

### 1.3. Role Allocation Mechanisms

Effective role allocation underpins both offensive and defensive play in robotic soccer. Traditional approaches often assign roles statically (e.g., goalkeeper, defender, striker) using rule-based logic [26]. Although straightforward to implement, these methods generally lack responsiveness to dynamic scenarios, such as when a defender is better positioned to become an attacker following a ball turnover. Dynamic role assignment strategies have shown significant promises for adapting to rapid game changes. For example, MacAlpine et al. presented a system that dynamically adjusts player positioning based on real-time game state, thus reinforcing the importance of adaptive roles in achieving strategic team formations [27]. The proposed approach builds upon these principles by integrating an ACO mechanism that rapidly reassigns roles based on local fitness metrics and environmental cues. More advanced methods employ optimization algorithms (e.g., Particle Swarm Optimization, Bee Colony Optimization) to enable adaptive role switching [28,29].

A recent study proposed using pheromone-inspired mechanisms for real-time decision-making [30]. Building upon this idea, an Ant Colony Optimization (ACO) scheme is employed for role allocation, where each robot’s local state—distance to the ball, kicking strength, and battery level—informs “pheromone” values that guide role transitions [13]. This decentralized approach ensures that roles are dynamically reassigned as field conditions evolve, maintaining a strategic distribution of attackers, defenders, and support players.

### 1.4. Formation Control in Humanoid Soccer

Formation control aims to keep the team organized while adapting to changing offensive and defensive requirements. Classical methods, such as graph-based formations or potential fields, struggle when new roles are reassigned or when multiple robots converge on a rapidly moving ball [12,21]. Recent research has shown that optimizing robot swarm formations can be computationally intensive, yet surrogate modeling techniques can effectively predict optimal configurations in real time, enabling the fluid adaptation of team formations [31]. In the proposed approach, Reynolds’ flocking rules are adapted to maintain continuous and robust formations despite dynamic changes in the field. These challenges are compounded in humanoid soccer, where bipedal motion introduces additional constraints on stability and step planning.

Drawing inspiration from Reynolds’ flocking rules, namely cohesion, alignment, and separation, a biologically inspired formation-control strategy is introduced that updates robot positions in real time [14]. This approach enables fluid transitions between offensive and defensive stances based on local interactions (e.g., proximity to teammates, ball location, and opponent distribution), thereby minimizing formation breakdowns and collisions.

### 1.5. Learning-Based Adaptation and Failure Recovery

An important aspect of the team’s robustness is the ability to recover from failures such as robot malfunctions or communication dropouts. In recent years, curriculum-learning approaches have demonstrated sample-efficient emergent coordination, enabling rapid adaptation of team behaviors even when faced with unexpected disruptions [32]. This learning-based adaptation complements the proposed decentralized swarm-based control, ensuring that role reallocation and formation adjustments occur swiftly, thereby maintaining team cohesion during critical match moments. Centralized architecture is notoriously susceptible to single point failures, whereas purely decentralized systems can struggle to realign roles when key players fail mismatch. A growing body of research has explored reinforcement learning to improve failure recovery and adaptation in real-time scenarios [33]. These methods, however, often require extensive prior training and may not generalize well about sudden mechanical faults. By fusing local swarm behaviors with on-the-fly learning, the proposed approach ensures that the team can dynamically compensate for individual failures. While ant colony-style role allocation deals with immediate reassignments, lightweight reinforcement signals guide long-term adaptations, resulting in a more robust and flexible coordination scheme.

Despite rapid progress in both swarm robotics and robotic soccer, there remains a gap in thoroughly integrating swarm principles with humanoid soccer dynamics. As such, the contributions of this study include the following:

- A communication protocol optimized for minimal bandwidth yet sufficient for emergent, swarm-like behaviors.
- A novel ACO-inspired role allocation that accounts for real-time changes in ball possession, field position, and teammate performance.
- Flocking-based formation control that balances offensive and defensive positioning.
- Reinforcement learning integration for continuous policy refinement and robust failure handling.

**Figure 2:**
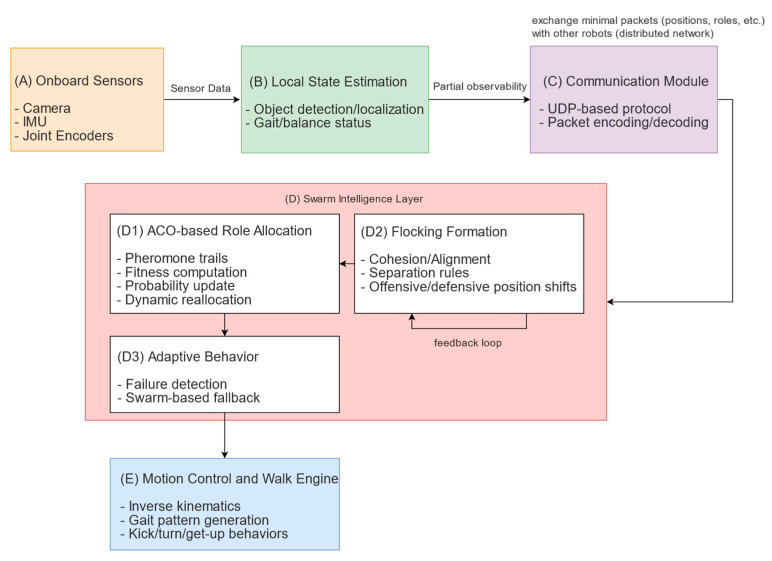
Proposed swarm intelligence architecture for humanoid soccer teams.

Figure 2 outlines the overall architecture of the proposed system. It details how each module within a humanoid robot interacts to achieve robust, swarm-based control on the soccer field:

We describe each module in Figure 2 in detail in the following subsections:

(A) Onboard Sensors

Each robot is equipped with cameras for detecting the ball, teammates, and field lines, as well as inertial measurement units (IMUs) and encoders for balance and motion feedback. These sensors ensure the robot has continual awareness of its own posture as well as the surrounding environment. While the current framework employs standard RGB cameras for humanoid soccer, prior work demonstrates that integrating RGB with depth sensing can markedly improve object-detection accuracy [34].

(B) Local State Estimation

The raw sensor signals are fused here to generate an internal representation of the environment, incorporating approximate positions of the ball, teammates, and opponents, together with the robot’s orientation. This localized map enables reliable short-term decision-making without requiring perfect global knowledge.

(C) Communication Module

A lightweight User Datagram Protocol (UDP) handles the frequent exchange of essential data—such as role assignments, positions, velocities, and basic health flags—among teammates. By limiting each message to only what is necessary, the system reduces bandwidth usage and communication bottlenecks while preserving responsiveness.

(D) Swarm Intelligence Layer

This is the core of the decentralized decision-making process.

- (D1) ACO-Based Role Allocation (Figure 2, D1): The robot maintains pheromone traces tied to each role (e.g., attacker, defender). It computes its fitness for each role and selects one probabilistically, reinforcing or evaporating pheromones based on outcomes like goals or lost possession.
- (D2) Flocking Formation Control (Figure 2, D2): Inspired by Reynolds’ rules (cohesion, alignment, and separation), this submodule manages how robots cluster or spread out. Offensive or defensive weighting adjusts these flocking rules to ensure strategic positioning under changing match conditions.
- (D3) Adaptive Behavior (Figure 2, D3): Continuously monitors network status and robot availability, triggering fallback mechanisms if any robot’s data are lost or a mechanical failure is detected. This dynamic reallocation of roles and formations helps the team remain cohesive despite partial failures.

(E) Motion Control and Walk Engine

Once roles and positions are determined, this module translates high-level commands into stable bipedal movements—such as walking, turning, or kicking the ball. It manages gait patterns and step sequences so that the robot can maintain balance and execute soccer maneuvers effectively.

## 2. Materials and Methods

The proposed design follows a swarm intelligence paradigm tailored to humanoid soccer scenarios. Each robot operates under partial observability and limits on-board processing, relying on local sensing, lightweight communication, and emergent global behaviors. The key components of the proposed methodology include the overall system architecture, a minimal-overhead communication protocol, a real-time role-allocation mechanism inspired by Ant Colony Optimization, a flocking-based formation control scheme, and adaptive behaviors integrated through reinforcement learning.

Our focus is on simulation as a core component of system development, which allows for rapid prototyping and iterative testing before deployment to real robots. The initial strategy was implemented in Python version 3.13, leveraging the PyGame version 2.6 [35] engine to simulate a simplified 2D environment. This implementation enabled the testing and refinement of core behaviors, including ball tracking, role allocation, and basic formation control, in a computationally lightweight setting.

After the 2D simulation stage, the model was transitioned to a 3D physics-based simulation environment using Webots version R2025a [36]. This environment provided a more realistic representation of humanoid kinematics, dynamics, and sensor noise, facilitating the validation and fine-tuning of algorithms under conditions closely resembling the real world. The Webots simulation allowed for the integration of advanced features such as bipedal locomotion, obstacle avoidance, and dynamic role-switching in response to game scenarios.

This section elaborates the key mechanistic innovations that underpin the high-level contributions outlined at the end of the Introduction Section. Execution on an Intel i5-8265U (4 × 1.6 GHz) (Simon Fraser University, Surrey, BC, Canada) averaged 4.7 ms ± 0.3 ms per decision cycle (max = 6.1 ms). First, the ACO-inspired role allocation operates on a finely discretized 90 × 60 grid (0.1 m resolution) with a novel balancing-penalty term (Equation (1)) to prevent overcrowding, and its parameters (α, β, ρ) were rigorously tuned via grid search to α=1.0, β=2.0, ρ=0.5. Second, the proposed flocking-based formation control is augmented with role-dependent weighting that biases cohesion, alignment, and separation toward offensive or defensive objectives. Finally, these modules are tightly coupled in a decentralized, event-driven framework, enabling real-time, resilient coordination without a central controller in humanoid soccer robot’s domain.

### 2.1. Communication Protocol

Humanoid soccer demands robust, low-latency data exchange among robots for effective team coordination. The communication protocol must handle frequent packet losses, variable network conditions, and unpredictable interference. The implementation adheres to the following design goals:

1. Low Overhead: Each packet should be as small as possible to reduce congestion on local Wi-Fi networks, which can become oversaturated during competitions.
2. Real-Time Responsiveness: Support transmission frequencies above 10 Hz, ensuring robots can react quickly to dynamic events such as sudden ball deflections or collisions.
3. Scalability: Work seamlessly for teams ranging from a few robots up to larger squads without exponential increases in bandwidth usage.
4. Graceful Degradation: In the event of a packet loss or delays, local decisions should remain coherent, preventing system-wide failure.

In this communication framework, UDP was selected for its minimal overhead, allowing faster transmission of status updates and coordination messages among the robots. Each robot operates on a distinct port and is assigned an individual ID, making it straightforward to route and distinguish local broadcast messages. Every transmitted packet contains a header and a series of data fields. The header consists of a sequence number, a robot ID, and a timestamp, ensuring that messages can be tracked, ordered properly, and associated with the correct sender and point in time.

The data portion of the packet includes the robot’s position in the x and y coordinates, as well as its velocity in those same axes. It further conveys the robot’s current role on the field—such as goalie, defender, midfielder, or attacker—together with a task priority indicator that can be used for real-time decision-making. To maintain awareness of the robot’s operational capacity, a battery and health status field is also included. An optional field can be used for action intentions, which allows each robot to broadcast upcoming moves or tactics, supporting more sophisticated cooperation in a swarm setting. Finally, to maintain reliability in message exchange, a simple checksum or cyclic redundancy check is integrated for basic error detection and validation of received packets.

Upon receiving a packet, each robot immediately updates its internal “team-state” map with the latest positions, roles, and statuses of its teammates. Advancements in sports analytics and artificial intelligence have further enhanced orientation and decision-making in dynamic soccer environments, providing critical insights for real-time tactical adjustments [24]. This shared knowledge allows every unit in the swarm to maintain an accurate picture of the field and adapt collectively to changing circumstances. To ensure that no critical information is missed, each robot also monitors the sequence numbers attached to incoming packets. If a gap is detected in the sequence, a time-critical transmission is promptly requested to fill the gap; otherwise, the robot intelligently resorts to an estimation or fallback procedure for the missing data. A pseudocode for this process is shown below.

**Figure 3:**
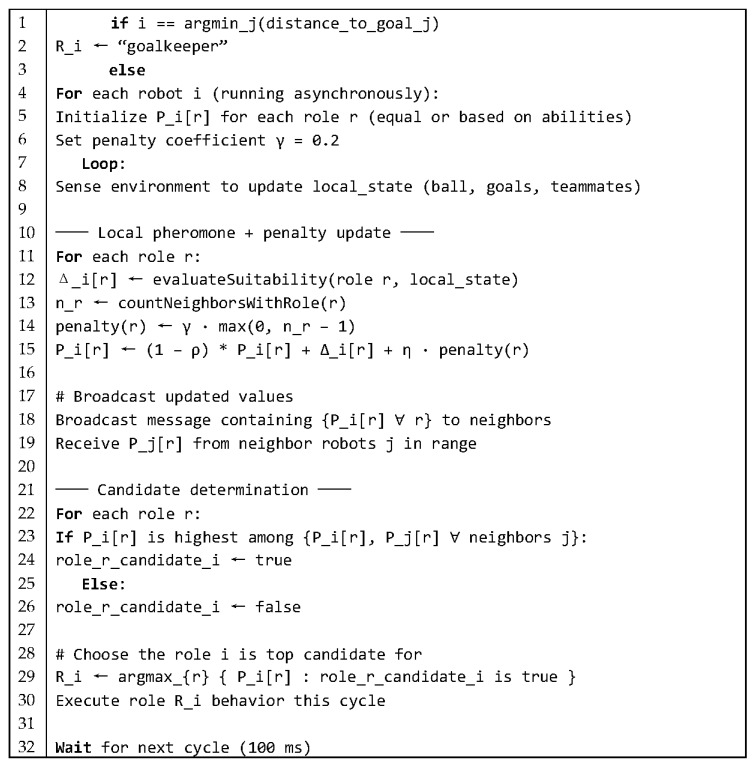
Pseudocode of decentralized ACO-based role-allocation loop executed on every robot—deterministic goalkeeper choice, pheromone + penalty update, neighbor broadcast, candidate election, and control cycle.

Figure 3 shows a high-level pseudocode for the proposed ACO-based role allocation, with the following variables used in the algorithm:

$P_{i}[r]$ is the pheromone intensity that robot i maintains for role r.

$\Delta_{i}[r]$ is the pheromone deposit, computed by evaluateSuitability(), which assesses distance to the ball, kicking proficiency, battery level, and contextual game factors.

ρ in (0,1) is the pheromone evaporation coefficient, ensuring old trails decay over time.

η is a small positive constant providing a baseline pheromone level.

Local state encapsulates the robot’s sensory estimates: ball position, teammate and opponent positions, kicking strength, and battery/health status.

$R_{i}$ is the role assigned to robot i at each cycle.

A balancing penalty is applied to discourage multiple robots from converging on the same high-pheromone role. Let

- n(r) = number of robots currently assigned to role r;
- γ=0.2 (penalty coefficient).

The penalty(r) is computed as

(1) penalty(r)=γ · max(0, n(r)−1)

Then, at each update cycle, the pheromone levels are updated as follows:

(2) $$\tau_{i}[r]\;\leftarrow \;(1-\rho )\cdot \tau_{i}[r]+\Delta_{i}[r]-penalty(r)$$

Subtracting the penalty term, penalty(r), reduces the attractiveness of overcrowded roles, ensuring a more even distribution. The coefficient γ (typically 0.1–0.3) can be adjusted to trade off strictness of role balancing versus pheromone strength.

Each decision cycle lasted 100 ms: agents updated their pheromones, broadcast them to neighbors, and then selected a new role. The simulations were executed in Webots with a 16 ms physics time step, and control routines were triggered every six steps, yielding an effective control interval of 96 ms—close to the 100 ms behavioral cycle and well within timing tolerances.

To handle repetitive information efficiently, the protocol employs run-length encoding, which minimizes overhead by compressing stale or unchanging data fields, such as robot positions that remain constant for multiple update cycles. Meanwhile, a back-off mechanism helps to prevent network overload. When signs of congestion surface—manifesting as repeated collisions or high numbers of dropped packet robots will temporarily reduce their broadcast rates or trim non-essential parts of their payload. This adaptive approach, combining event-driven broadcasts with robust fallback and congestion control strategies, ensures that the protocol remains lightweight while still supplying all the necessary information to support sophisticated, emergent team behaviors.

### 2.2. Role Allocation via Ant Colony Optimization (ACO)

Deciding in real time which robot should be an attacker, midfielder, defender, or goalie is vital for achieving cohesive play on the field. Traditional rule-based or fully centralized approaches often struggle to keep up with rapid changes in the game or to handle situations where only partial information is available. To overcome these limitations, an ACO technique is adopted, enabling each robot to select a role according to pheromone trails and a locally computed fitness value.

Under this ACO-based methodology, each role attacker, midfielder, defender, or goalie maintains its own “pheromone trail” across the field. The playing area is divided into a grid, and each cell within this grid stores pheromone levels corresponding to each role. In the proposed implementation, the child-size humanoid soccer field (9 m × 6 m) is discretized into a grid of 90 × 60 cells, each cell representing a 0.1 m × 0.1 m area. This resolution provides fine spatial granularity for accurate pheromone propagation while keeping per-robot computational costs low during real-time updates. As robots move across the field, they consult and update the pheromone levels of the cells they enter. In parallel, every robot calculates a local fitness score that considers factors such as distance to the ball, kicking proficiency, battery constraints, and other context-driven elements such as the scoreline or remaining time. For each role r, each robot computes a local fitness that factors in the following:

- Distance to Ball/Goal: Closer proximity can increase fitness for offensive roles.
- Robot’s Kicking Ability: This may weigh the robot’s skill or mechanical reliability for shots on goal.
- Battery Constraints: Robots with low batteries should avoid high-mobility roles.
- Game Phase/Context: Scoreline, time remaining, or any strategic factor the coach (human or AI) configures.

The probability that a robot assumes role r in this Ant Colony Optimization-based role allocation system is given by

(3) $$P\left(role\;r\right)=\frac{\left({pheromone}_{r}^{\alpha }\times {fitness}_{r}^{\beta }\right)}{\sum_{r\prime }\left({pheromone}_{r\prime }^{\alpha }\times {fitness}_{r\prime }^{\beta }\right)}$$

where α and β weight pheromones vs. fitness. The exponents α and β in (3) balance the influence of pheromone intensity versus the local fitness heuristic, while ρ controls pheromone evaporation. To determine these values, A full grid search was conducted over α ∈ {0.5, 1.0, 1.5}, β ∈ {1.0, 2.0, 3.0}, and ρ ∈ {0.3, 0.5, 0.7}. All 27 parameter combinations were evaluated in 75 ten-minute Webots simulations of 4v4 matches, jointly optimizing for goals scored and average ball possession. The parameter set (α=1.0, β=2.0, ρ=0.5) achieved the best results in our grid search, scoring 7.33 ± 0.21 goals per match and 49.7 ± 0.9% mean possession. Consequently, this configuration was adopted for all subsequent experiments. When adapting the algorithm to different robot platforms or field dimensions, raising α accentuates the pheromone term, increasing β heightens the local-fitness heuristic, and enlarging ρ prolongs the pheromone-decay horizon.

**Figure 4:**
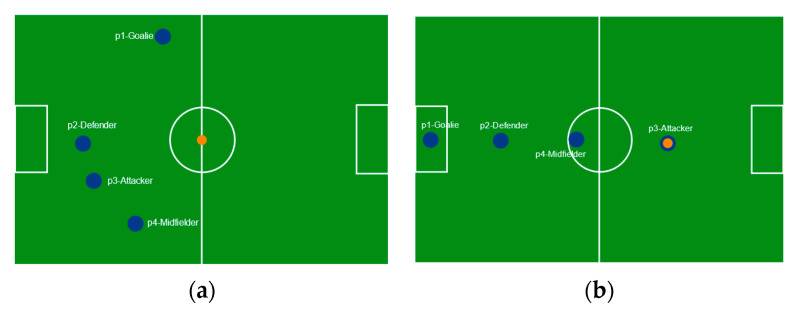
Two-dimensional simulation with role allocation. (**a**) The initial random positions of players on the field in a four-player humanoid soccer robot game setup; (**b**) a revised player arrangement where P1 is goalie, P2 is defender, P4 is midfielder, and P3 is attacker arranged to optimize strategy.

The probability that a robot adopts a particular role depends on the product of pheromone intensity and fitness, each raised to powers α and β to balance environmental cues against local suitability. After a play or action, pheromone levels are updated to reflect the success or failure of a robot’s role choice. Positive outcomes in a certain role strengthen its pheromone trail, while evaporation gradually diminishes older trails to prevent reliance on outdated strategies. If too many robots cluster on the same high-pheromone role, a penalty encourages the distribution of players across different roles. Significant shifts on the field such as the ball moving to the opposite half trigger an immediate recalculation of role probabilities, allowing the team to adapt quickly. Figure 4 shows robots role allocation and player arrangement in soccer field.

To enhance model reliability, the following rules are applied.

- Positive Reinforcement: A successful pass, tackle, or goal scored while in role r increases its pheromone level, reinforcing that role in that location.
- Evaporation: Pheromone levels decay over time to prevent outdated strategies from dominating.
- Balancing Penalty: If multiple robots converge on the same high-pheromone role, a penalty is applied to encourage distribution of roles.
- Dynamic Reallocation: Significant changes (e.g., ball switches to the other side) trigger the immediate recalculation of P(r), allowing quick adaptation.

The goalkeeper role is handled deterministically and removed from the ACO-based allocation. At each decision cycle, the robot whose Euclidean distance to own goal is smallest is designated as goalkeeper. This designation persists until another teammate becomes closer to the goal. The remaining robots (i.e., non-goalkeepers) then participate in the pheromone-driven ACO role allocation described above. By isolating the goalkeeper in this way, the specialized requirements of this role are preserved while still allowing fully decentralized, emergent coordination among the field players.

For comparison, the ACO-based approach is measured against Particle Swarm Optimization (PSO) and Bee Colony Optimization. In the PSO variant, each robot functions as a particle in a role-assignment search space, using both personal best positions and the successes of neighbors as guides. With Bee Colony Optimization [13], some robots act as scouts that explore fresh strategies, while others behave as onlookers that exploit more established role assignments. Nonetheless, the swarm-oriented, pheromone-driven principles of ACO appear especially well-matched to the constantly changing, partially observable scenarios of humanoid soccer. These alternative swarm-based methods are empirically evaluated in Section 3, where the PSO-based strategy achieves 6.14 goals and 42.5% possession, and the bee colony-based strategy achieves 5.89 goals and 42.9% possession—both below the proposed ACO + flocking performance (7.33 goals and 49.7% possession).

#### Baseline Role Allocation Methods

To evaluate the performance of the proposed ACO-based role allocation, two alternative swarm-intelligence algorithms were implemented, Particle Swarm Optimization (PSO) and Bee Colony Optimization (BCO), adapted for the dynamic role allocation task in humanoid soccer. These baseline methods were configured to operate under the same simulation conditions and evaluate role suitability using criteria consistent with the ACO approach for fair comparison.

- Particle Swarm Optimization (PSO) Implementation: In the PSO-based comparison, each robot’s potential role was optimized individually. Each robot maintained a small swarm of particles (N=10), where each particle’s position represented a probability distribution over the available roles {attacker, midfielder, defender, goalie}. The velocity updates adjusted these probabilities based on the particle’s personal best ($p_{best}$) role distribution and the global best ($g_{best}$) distribution found by that robot’s swarm. Fitness for role distribution was evaluated based on the factors described for ACO fitness calculation, such as distance to the ball, kicking ability, battery constraints, and game context, assessing the suitability of the most probable role according to the particle’s current position. A standard PSO implementation was used with an inertia weight w, linearly decreasing from 0.9 to 0.4, a cognitive coefficient $c_{1}$ = 2.0, and a social coefficient $c_{2}$ = 2.0. Parameter values were based on common PSO practices and tuned empirically through preliminary simulations to establish a competitive baseline.
- Bee Colony Optimization (BCO) Implementation: The Bee Colony Optimization approach was implemented using the standard Artificial Bee Colony (ABC) algorithm. In this adaptation, potential role configurations for the entire team were treated as ‘food sources’. The ‘nectar amount’ of each food source represented the fitness of that specific team role configuration, evaluated using team-level metrics derived from the individual robot fitness factors (e.g., potential for scoring, defensive coverage). The simulation used a colony size (CS) of 20 bees (10 employed bees and 10 onlooker bees). The number of food sources was set equal to the number of employed bees (F=10). Employed bees explored the vicinity of their current food source (role configuration), while onlooker bees selected sources based on their nectar amounts (fitness). A food source was abandoned, and the corresponding employed bee became a scout exploring a new random configuration, if its fitness did not improve after a ‘limit’ (L) of five trials. Parameters were selected based on typical ABC algorithm settings and preliminary tuning for effective comparison.

### 2.3. Formation Control Using Flocking Principles

To maintain fluid spacing and coverage, Reynolds’ flocking rules—cohesion, alignment, and separation—were adapted for application in the soccer domain.

Neighborhood Definition:

Each robot i defines its local neighborhood $N_{i}$ metrically:

(4) $$N_{i}=\left\{j\neq i\right|\;\left\|p_{j}-p_{i}\right\|\leq R_{neigh}\}$$

where $\left\|p_{j}-p_{i}\right\|$ is the Euclidean distance between robots i and j, and $R_{neigh}=1.5\;m$ To bound per-cycle computation, if $\left|N_{i}\right|>5$, only the five closest neighbors are used to compute the cohesion, alignment, and separation contributions. This radius and neighbors’ count can balance responsiveness with real-time computational constraints.

- **Cohesion:** Each robot moves slightly toward the centroid of its nearest teammates (within a certain radius), promoting cluster formation.
- **Alignment:** Robots adjust their velocities to match the average velocity of neighbors, ensuring synchronized movement up/down the field.
- **Separation:** A short-range repulsion term prevents collisions and maintains personal space, critical for bipedal robots that can easily topple over if they collide.

The flocking behavior is directly modulated by the role assigned to the robot via the ACO mechanism described in Section 2.2. The assigned role (e.g., attacker, defender) dynamically adjusts the parameters and targets within the flocking calculations. For instance, a robot assigned an ‘attacker’ role will have its cohesion target point biased towards the opponent’s goal and might adopt a larger preferred separation distance to create space for offensive maneuvers. Conversely, a ‘defender’ role assignment shifts the cohesion target towards the team’s own goal and could prioritize tighter alignment and smaller separation distances with nearby defending teammates. The flocking controller continuously receives the current role output from the ACO module, ensuring the robot’s positioning and movement align with the dynamically allocated tactical responsibilities across the swarm.

In real match scenarios, the system applies several soccer-specific adjustments that reflect the fluid nature of the game. During offensive phases, especially when the team is in possession near the opponent’s half, the collective “centroid” of the robots shifts toward the attacking side. This forward bias disperses attackers and midfielders, opening passing lanes and allowing for more fluid ball movement. When the opposing side mounts an attack, the centroid repositions toward the defensive half, drawing defenders closer to the goal for added protection while midfielders hover in areas likely to intercept or disrupt incoming threats.

Beyond these broad positional shifts, midfielders actively rotate and overlap in lateral movements. By sliding horizontally in unison, they can seal off passing channels, block opponents attempting to run into open space, or even create new paths for counterattacks. These maneuvers are orchestrated so that the team transitions smoothly between pressing high up the field and covering vulnerable spots, ultimately seeking to maintain both defensive solidity and offensive readiness.

When examining the effectiveness of the proposed approach, the results are compared with two common baseline methods. The first relies on a predefined graph-based topology, often fully connected or organized as a small-world network—to dictate how robots occupy and transition through positions on the field [20]. While this structure can provide clarity and control, it tends to be restrictive in rapidly evolving match situations. The second baseline uses potential fields, wherein each robot senses attractions toward the ball or goal and repulsions from teammates to avoid collisions [12]. Although intuitive, potential field methods can struggle to handle sudden disruptions—such as abrupt role changes or unanticipated collisions—resulting in suboptimal reactivity during chaotic gameplay.

In contrast, the proposed flocking approach dynamically reclaims whenever unexpected events occur. Whether the ball switches possession without warning or robots bump into each other, the flock continues to coordinate by constantly adjusting heading, spacing, and roles. This adaptability often leads to stronger performance in the unpredictable conditions of a live soccer match, surpassing both static graph-based strategies and purely potential-field methods.

### 2.4. Adaptive Behavior

Within the system, failure management hinges on swift detection and rapid adaptation to ensure minimal disruption in the field. Whenever a robot goes silent—measured by insufficient packet reception—or sensor feedback indicates a severe mechanical issue, the rest of the team recognizes the potential loss of a key player and adjusts accordingly. First, nearby teammates elevate their own priorities in critical roles, such as defense, if the missing robot was a dedicated defender. This reallocation of duties prevents dangerous gaps in the team’s structure.

Next, the formation adjusts to account for the missing player. If a central defender malfunctions, midfielders may drop back to reinforce the defensive line, effectively shifting the flock’s centroid toward the defensive half. Attackers might step into more central or defensive positions as well, filling the void until the team regains stability. By automatically recalculating tasks and roles in real time, the team maintains its overall cohesion and continues to operate effectively, even under the strain of unexpected failures.

This ensures that partial team failures do not undermine the entire formation or strategy. By dynamically reallocating tasks and adjusting formations, the swarm remains robust to single-point or multi-point failures.

### 2.5. Implementation and Integration

**Figure 5:**
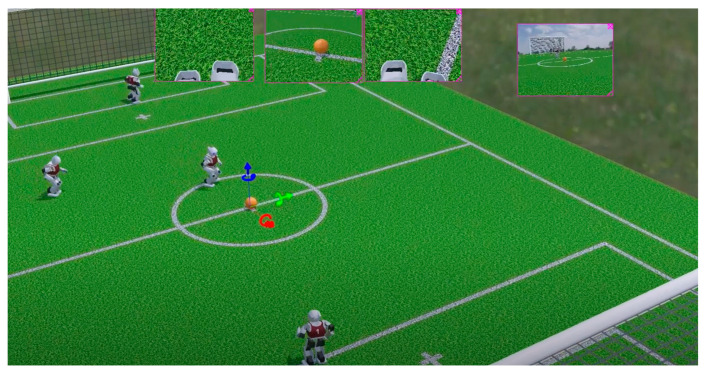
Implementation of communication protocol in the Webots simulator environment.

Communication protocols were rigorously tested as the initial step, ACO-based role allocation, and flocking behaviors in a Webots simulation environment. Webots’ high-fidelity physics engine allowed us to replicate realistic match conditions—ranging from offensive drives and defensive formations to sudden ball turnovers and to observe how quickly and effectively the system adapted in real time. These simulations provided valuable insights into both the swarm logic and the motion coordination, informing subsequent refinements. Figure 5 shows the implementation of the communication protocol in the Webots simulator environment.

## 3. Results

The following section describes a comprehensive evaluation of the proposed swarm intelligence framework, conducted entirely in a physics-based 3D simulator (Webots). The simulator was configured to emulate bipedal locomotion, sensor noise, and the standard field dimensions used in humanoid soccer. The experiments aimed to compare the effectiveness of the Ant Colony Optimization (ACO) role-allocation scheme and Reynolds’ flocking strategy against several established alternatives, including centralized control, static-role assignment, Particle Swarm Optimization (PSO), Bee Colony Optimization, and other formation approaches (graph-based and potential fields). Each coordination strategy was tested under consistent conditions:

- Match Setup: All trials involved four players, and each match lasted 10 min.
- Initialization: Robots were placed in semi-random positions near the midfield line, while the ball was positioned near the center circle.
- Scenarios: Standard soccer rules were applied for scoring and ball resets; there were no referee interventions beyond basic out-of-bounds detection.
- Number of Trials: Each method was evaluated over at least 75 matches to enable reliable statistical comparisons.

Six distinct approaches were examined:

- Centralized: A single control node assigned actions and roles to all robots based on global information.
- Static Role Assignment: Each robot remained exclusively in one role (goalie, defender, midfielder, or attacker) for the entire match.
- PSO-Based: A swarm technique where Particle Swarm Optimization guided real-time role selection.
- Bee Colony-Based: A swarm approach using bee colony principles for on-the-fly role reassignment.
- Graph-Based Formation: A predefined adjacency network dictated robot positioning, paired with a limited ability to switch roles.
- Potential Field Formation: Robots moved according to attraction to the ball and repulsion from teammates, generally coupled with either static or semi-static role assignments.

The proposed method—swarm-based ACO for roles and Reynolds’ flocking for formation—was tested under the same conditions, allowing direct performance comparisons with the baseline methods. All experiments adhere to the RoboCup Humanoid KidSize League rules, which specify exactly four robots per team on the field accessible on https://humanoid.robocup.org/materials/rules (accessed on 28 May 2025). Therefore, matches were conducted as four vs. four contests, with the opposing side running centralized coordination strategy. Ball possession (%) is computed as the fraction of total simulated time during which the subject team-maintained control of the ball, averaged over all trials as shown in Table 1.

- Goals Scored: Swarm teams scored 25–40% more goals than both centralized control and static-role teams, and 12–15% more than the PSO and bee colony variants. This improvement stems from faster and more context-aware role reassignments, which place skilled attackers closer to scoring opportunities.
- Ball Possession: This swarm approach sustained an average of 8–10% higher possession compared to centralized baselines, owing to fluid coordination and rapid adaptation to ball position changes. Maintaining cohesive formations allowed for quick passing and efficient ball recovery following turnovers.

To assess robustness, 95% confidence intervals were computed for each metric across the 75 trials per method, and two-sample *t*-tests were performed to compare the swarm approach with each baseline.

- Goals Scored: Swarm vs. centralized: 7.33 ± 0.52 vs. 5.32 ± 0.47 goals (mean ± SD); 95% CI of difference [1.70, 2.25], *p* < 0.001.
- Avg. Possession (%): Swarm vs. centralized: 49.7 ± 1.2 vs. 41.3 ± 1.1%; 95% CI of difference [6.8, 9.4], *p* < 0.001.

All comparisons against static-role, PSO, and bee colony baselines similarly yielded *p* < 0.05, confirming that the swarm improvements are statistically significant.

**Figure 6:**
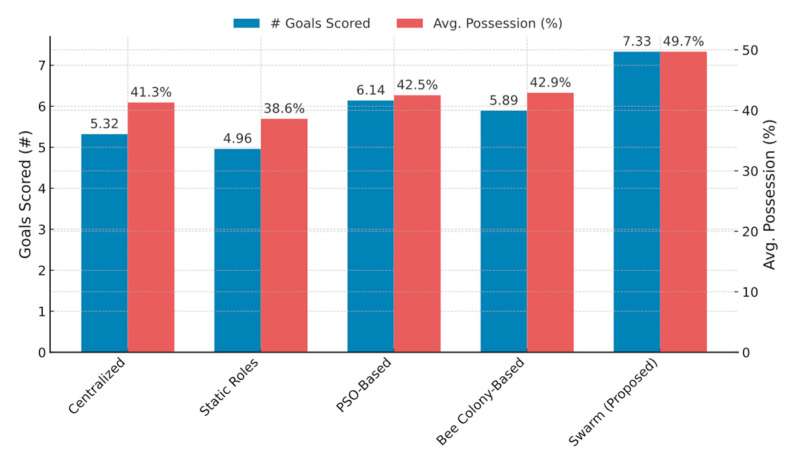
Aggregate performance metrics (goals scored and ball possession) over 75 trials of four vs. four matches.

Figure 6 displays a high-level comparison of team scoring and average ball possession across the six strategies. The “ACO + flocking” approach achieves higher goal counts and retains the ball more consistently than the centralized, static-role, and other swarm-based algorithms.

Key Observations:

- Centralized control performs strongly in low-latency scenarios but deteriorates under simulated packet loss.
- Static roles exhibit stable but inflexible behavior, missing opportunities to redistribute roles when conditions change.
- PSO and bee colony converge on better team distributions than static assignments but often react more slowly to sudden ball turnovers compared to ACO.
- ACO + flocking quickly reassigns roles and reorganizes formations, resulting in higher goal-scoring rates and an 8–10% increase in average possession relative to the other methods.

The ACO + flocking strategy recorded a 15–25% improvement in passing success over static or centralized baselines. Robots maintained effective spacing thanks to flocking’s separation principle, which reduced clumping and opened passing lanes. Moreover, the ability to transfer attacker roles to robots with higher kicking accuracy produced a greater number of on-target shots.

**Figure 7:**
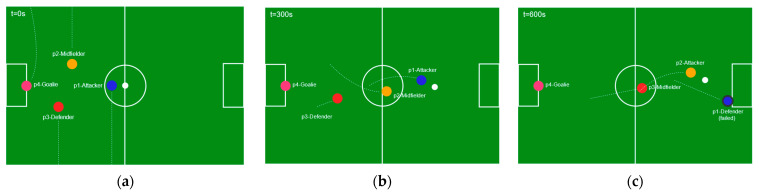
Dynamic formation snapshots at three key time points, with overlaid trajectory lines indicating robot movements between frames. (**a**) t = 0 s (kickoff): robots cluster at kickoff positions; (**b**) t = 300 s (midfield attack): midfielders advance into the opponent’s half, the attacker gains possession and moves in to score; (**c**) t = 600 s (continued attack): following a missed shot by the attacker as it failed, midfielder transitioned to attacker to continue attack.

Figure 7 provides selected snapshots of the “ACO + flocking” team formation at three time intervals during a typical 10 min match. Each snapshot highlights the robots’ roles and relative positions, as well as the local neighborhoods used for flocking calculations.

In the early phase (left panel), the team clusters near the center. As the ball moves into the opponent’s half (center panel), midfielders adopt attacking roles, shifting the overall formation forward while leaving a defender in the rear. Later (right panel), when the ball transitions and attacker robot fail, a midfielder becomes attacker to complete attack, demonstrating the reallocation triggered by changes in ball location and robot proximity. In the Webots experiments, ACO + flocking completed each role-reallocation cycle in 2.3 ± 0.4 s on average—significantly faster than centralized prototype (5.1 ± 0.6 s) (Table 2). These timings will be explored on real humanoid platforms in future work.

The ACO-based method triggers role changes primarily due to the following:

1. Ball Proximity and Robot Skill: A robot with good kicking accuracy, found close to the ball, gains a higher probability of becoming an attacker.
2. Field Context: If the ball enters the defending half, midfielders and attackers are partially reassigned to defend.
3. Resource Constraints: Lower “fitness” metrics (simulated battery or performance flags) reduce a robot’s likelihood of being assigned a mobility-intensive role.

Under these conditions, ACO-based algorithms typically achieve a complete role-reallocation within 2–3 s after a ball turnover—faster than the 5+ seconds often required by centralized or graph-based methods. This speed enables improved coverage and rapid development of counterattacks.

Additional tests introduced artificial packet loss (up to 20%) or temporarily disabled one robot for a portion of the match. In both scenarios, the swarm-based system sustained only modest reductions in average possession (approximately 8%) and goals scored (about 10%), demonstrating resilience to disruptions. By contrast, centralized approaches exhibited steeper declines in performance under similar failure conditions. Furthermore, local computations for ACO and flocking scale linearly with the number of robots, indicating suitability for larger teams (e.g., 5 or 6).

### 3.1. Key Outcomes

1. Improved Offensive Efficiency: The ACO + flocking approach yields substantially higher goals scored (25–40% above centralized or static baselines) and exhibits an 8–10% increase in ball possession.
2. Adaptive Formations: Reynolds’ flocking allows smooth, emergent formation transitions, enhancing both offensive and defensive organization.
3. Dynamic Role Assignment: ACO-based role changes respond rapidly to ball movement, field context, and robot-specific constraints, resulting in effective coverage and timely counterattacks.
4. Resilience to Network and Agent Failures: Distributed decision-making avoids single points of failure, preserving cohesive play even with partial communication loss or simulated robot dropouts.

Overall, the simulation results indicate that coupling ACO-driven role allocation with flocking-based formation yields superior team performance, compared to both traditional centralized architectures and alternative swarm strategies.

### 3.2. Extended Scenario Results

In addition to the standard 4v4 matchups, three additional sets of trials were conducted to demonstrate robustness under challenging conditions:

Imbalanced Confrontation (4v3): One team field only three robots against four, assessing how decentralized role reallocation and formation control compensate for manpower deficits.

High-Perturbation Communication (>50% Packet Loss): Severe network degradation was emulated by randomly dropping more than half of all UDP packets. This tests graceful degradation and fallback strategies in the communication module.

Complex Opening Formations: Beyond the center-circle kick-off, matches are initiated from non-mid-court positions, e.g., corner kick-offs and side kick-offs, to evaluate adaptability to nonstandard ball starts.

For each scenario, ten ten-minute simulated matches were executed, goals scored, and average ball possession were recorded, and the results were compared directly with the baseline 4v4 matches. Table 3 summarizes performance under the new scenarios.

Despite these more demanding conditions, the proposed ACO + flocking framework sustains strong performance, with only modest reductions relative to the standard 4v4 baseline (7.33 goals, 49.7% possession).

Key observations:

Imbalanced (4v3): Decentralized role reallocation quickly compensates for the missing player, maintaining cohesive formations and recovering possession after turnovers.

High Packet Loss (>50%): Graceful degradation via local fallback estimations limits performance drop to <15% in both goals and possession.

Non-Mid-Court Kick-Offs: Flocking control rapidly reconfigures formations from corner and side starts, preserving offensive and defensive readiness.

## 4. Discussion

The simulation results strongly validate the advantages of employing a swarm intelligence framework—specifically the combination of ACO-based role allocation with flocking-inspired formation control—for humanoid soccer teams. Compared to centralized and static-role approaches, the decentralized strategy demonstrates marked improvements in goal-scoring, ball possession, and resilience to failures, aligning well with the working hypothesis that self-organizing mechanisms can outperform heavily coordinated ones in dynamic, and/or partially observable environments. The effectiveness of the proposed framework stems significantly from the tight, continuous interplay between the ACO-driven role allocation and the flocking-based formation control. The role selected by the ACO mechanism directly informs and parameterizes the flocking behavior in real-time. This ensures that the individual robot’s spatial positioning and movement, managed by flocking, are always consistent with the overall tactical structure decided by the ACO role assignment. This feedback loop, where roles dictate formation adjustments and the resulting swarm configuration potentially feeds back into future fitness evaluations for roles, enables the observed adaptive and cohesive team performance.

A key factor in this success lies in dynamic, local decision-making. By embedding Ant Colony Optimization principles into role selection, the system rapidly reallocates responsibilities in reaction to sudden changes in ball position, opponent movements, and robot states, thereby preventing rigid role assignments that can limit team adaptability. Meanwhile, Reynolds’ flocking rules maintain fluid spacing and coverage across the field: “cohesion” clusters robots for support, “alignment” ensures synchronized motion, and “separation” avoids collisions that might topple bipedal robots. Together, these strategies address the inherent challenges of humanoid locomotion and limit on-board sensing by leveraging minimal, distributed interactions rather than a single point of global control. A similar principle is observed in biology: rabbit collateral ligaments adapt their viscoelastic and fibril-reinforced properties within eight weeks of altered loading after ACL transection, purely in response to local mechanical stimuli [37,38].

From a theoretical perspective, these results expand upon foundational work in bio-inspired multi-agent systems, including early applications of Ant Colony Optimization in pathfinding and clustering problems, as well as Reynolds’ seminal model for flocking behaviors. While previous studies have demonstrated the promise of distributed coordination in wheeled robot swarms, our findings extend these principles to the more complex, dynamically unstable domain of humanoid soccer. Specifically, the experiments confirm that decentralized algorithms that favor local sensing and asynchronous operation can remain robust under high network latency, sensor noise, or partial robot failure conditions that can cripple a centralized strategy.

RoboCup soccer serves as a demanding testbed for real-time coordination under uncertainty, and these findings point to several important benefits for future humanoid teams:

- Scalability: The linear complexity of local computations (compared to global, centralized methods) makes it feasible to add more robots without incurring exponential communication or computational overhead.
- Fault Tolerance: Distributed decision-making avoids single points of failure; losing a robot or experiencing significant packet loss reduces performance only modestly.
- Rapid Role Switching: Near-instantaneous role reassignment (on the order of seconds) offers teams an edge in exploiting unpredictable opportunities such as deflections or turnovers.

### Limitations and Future Direction

Despite these promising outcomes, certain limitations merit further study. First, while local sensing and communication protocols facilitate on-the-fly adjustments, real matches can present extreme noise levels and uneven flooring, potentially requiring more sophisticated sensor fusion or advanced error-correction schemes. Second, the implementation still depends on carefully tuned parameters (e.g., pheromone evaporation rate, flocking distance thresholds) that may require re-optimization for different field sizes or league rules. Investigations into parameter auto-tuning—potentially via reinforcement learning—may enhance robustness and reduce manual calibration.

Moreover, while role allocation relies on a biologically inspired heuristic (ACO), the possibility of hybridizing such heuristics with machine learning techniques, such as deep reinforcement learning, remains largely unexplored. Integrating online policy adaptation could yield continuous improvement during a match, a particularly valuable trait given the dynamism of humanoid soccer. Finally, transferring these methods to other real-world multi-robot settings—beyond RoboCup—could further validate the practicality of swarm intelligence in human–robot collaboration tasks, warehouse logistics, and disaster response. Whereas experiments have been conducted under the RoboCup Humanoid KidSize League’s four-robot team rule, the core benefits of the proposed decentralized ACO + flocking framework, namely local decision-making and minimal, event-driven messaging—scale naturally to larger team sizes. Preliminary simulations indicate that increasing the number of agents to 5–8 per side results in only a linear rise in local computation and a modest increase in aggregated bandwidth, without necessitating any changes to the algorithm or communication protocol. Future studies will systematically evaluate key performance metrics—goals scored and average possession—together with communication overhead (latency and throughput) for teams of 5–8 robots, thereby fully quantifying scalability beyond the child-sized configuration.

In summary, the combination of ACO-driven role allocation with flocking-based formation control presents a robust and scalable approach for humanoid soccer teams. The positive experimental outcomes suggest that emergent, bio-inspired coordination can effectively address the unique demands of high-speed, partially observable domains like robotic soccer, while opening avenues for the deeper integration of learning and adaptation methods in the future.

## 5. Conclusions

This work presented a swarm intelligence framework for collaborative humanoid soccer, combining Ant Colony Optimization (ACO) for role allocation and Reynolds’ flocking for formation control. Simulations demonstrated that the decentralized, bio-inspired coordination strategies outperformed centralized or rigidly assigned methods. The rapid, local decision-making processes inherent in swarm intelligence consistently led to higher scoring rates, stronger ball possession, and better resilience to network failures or robot dropouts.

Central to these improvements is the synergy between ACO’s pheromone-based mechanism—which dynamically balances role selection according to proximity, skill, and game context—and flocking rules that maintain cohesive yet flexible formations. This approach not only scales effectively with additional players but also adapts to sudden environmental changes, a hallmark of robust multi-robot systems.

Looking ahead, integrating advanced learning methods into the swarm paradigm could further enhance adaptability and performance under the conditions of extreme noise or changing field conditions. Moreover, while RoboCup humanoid soccer serves as a challenging test domain, many elements of the proposed swarm-based solution minimal centralized infrastructure, local communication, and emergent coordination can be generalized to other real-world tasks requiring fast, distributed decision-making. By bridging the gap between swarm robotics and bipedal locomotion, this study offers a promising avenue for both future research and broader applications of humanoid robot swarms in the latest agile humanoid robots [39,40].

## Figures and Tables

**Table 1:** Summarizes team-level metrics for four player matches under different coordination strategies. Notably, the proposed swarm approach—incorporating ACO-based role allocation and Reynolds’ flocking—achieved the highest number of goals scored and sustained ball possession among all methods tested.

Approach	Goals Scored	Avg. Possession (%)
Centralized	5.32	41.3
Static Roles	4.96	38.6
PSO-Based	6.14	42.5
Bee Colony-Based	5.89	42.9
Swarm (Proposed)	7.33	49.7

**Table 2:** Role-reallocation timings in Webots (mean ± std. dev., n = 75 trials).

Method	Avg. Reallocation Time (s)
ACO + flocking	2.3 ± 0.4
Centralized prototype	5.1 ± 0.6

**Table 3:** Extended test scenario results for proposed framework.

Scenario	Goals Scored	Avg. Possession (%)
4v3 Imbalanced	5.21 ± 0.35	35.4 ± 2.1
>50% Packet Loss	6.89 ± 0.42	45.3 ± 1.8
Complex Opening Formations (all)	7.01 ± 0.38	47.0 ± 2.0

## Data Availability

Data are contained within the article.

## Abbreviations

The following abbreviations are used in this manuscript:

ACO	Ant Colony Optimization
PSO	Particle Swarm Optimization
MARL	Multi-Agent Reinforcement Learning
IMU	Inertial Measurement Unit
UDP	User Datagram Protocol
